# A Preliminary Study of the Treatment Outcomes of Paediatric Dental Patients Referred for General Anaesthesia or Sedation at a Regional Hospital in Trinidad

**DOI:** 10.3390/children8100876

**Published:** 2021-10-01

**Authors:** Tricia Percival, Reshma Bhagoutie

**Affiliations:** School of Dentistry, University of the West Indies, St. Augustine, Trinidad and Tobago; reshmabhagoutie@hotmail.com

**Keywords:** paediatric dentistry, general anaesthesia, sedation, treatment outcomes

## Abstract

General anaesthesia and sedation are known to be useful adjuncts in the care of paediatric dental patients. There are several challenges that prevent patients from receiving care. Aim: To assess the treatment outcomes of paediatric dental patients seen at an emergency facility who were referred for treatment under sedation or general anaesthesia at a regional hospital in Trinidad. Methods: Records of patients seen at the Child Dental Health Unit Emergency clinic at the University of The West Indies Dental School from 2012 to 2017 were assessed. The parents of children referred for general anaesthesia or sedation at the regional hospital were then interviewed via telephone. Results: Most children (53.4%) were younger than 6 years old and the most common reasons for referral were the treatment of multiple carious teeth and behaviour management. Furthermore, 66.1% of cases did not receive treatment and had a mean waiting time of 4.7 years, and 61.7% of referred cases needed emergency care while awaiting general anaesthesia or sedation. Limited access to these services and the high cost of treatment were the main reasons for non-treatment. Conclusion: There is significant need for the timely treatment of paediatric dental patients referred for general anaesthesia or sedation. Improved availability and accessibility of these services could improve patients’ quality of life.

## 1. Introduction

In Trinidad and Tobago, children receive dental care through a variety of avenues. Dental treatment can be obtained through private practices or through government dental clinics at community health centres. However, in many instances, the treatment services offered at these facilities are determined by a patient’s co-operation or are limited to simple restorations and extractions. There is a high prevalence of dental caries, especially in young children, in Trinidad and Tobago [1,2], and for children who do not allow for treatment due to their young age, who exhibit limited cooperation or significant medical or physical challenges, adjunctive services such as dental sedation or general anesthesia (GA) are desired [3,4,5,6]. These services allow the dentist to provide a high quality of care to patients while minimizing dental anxiety [7].

Dental GA services include either the provision of exodontia only or comprehensive dental treatment under general anaesthesia. Dental sedation services include the use of oral, inhalation, or intravenous sedation to facilitate the provision of dental treatment. Currently, in Trinidad and Tobago there are two main avenues to access dental GA/Sedation services, either privately or at one of the regional hospitals of the Eric William Medical Sciences Complex (EWMSC). Other major government-owned health facilities located in the west, south, and east of Trinidad, and on the sister isle of Tobago, do not consistently offer the aforementioned services. Hence, all referred patients who cannot afford treatment privately depend heavily on the government health services provided by the EWMSC located in the North Central region of Trinidad.

The University of the West Indies School of Dentistry, Child Dental Health unit (CDHU) provides comprehensive oral rehabilitation and emergency dental care services under local anaesthesia (LA). Patients attending this unit are either self-referred or referred from private clinics or government health centres. Children who require adjunctive services are either referred for private specialist care or, as a public alternative, to EWMSC’s Oral and Maxillofacial Surgery (OMFS) department for treatment under GA. In some instances, it has been observed that patients re-attended the CDHU emergency clinic with repeated episodes of pain and/or courses of antibiotics due to reported delays in receiving care. This was found to be consistent with similar reports on delayed treatment under GA, affecting patient quality of life [8,9,10].

To date, there are no published data on the demand for adjunctive paediatric dental services such as sedation or GA and treatment outcomes in Trinidad and Tobago. The purpose of this study was to conduct a preliminary investigation on the treatment outcomes for patients in a paediatric dental emergency clinic who were then referred for dental treatment under GA/sedation at a regional hospital. This retrospective clinical audit was also carried out with the aim of identifying deficient areas of the current practice and thereafter to suggest ways in which one could improve services to provide a higher quality of clinical care to paediatric dental patients [11].

## 2. Materials and Methods

Permission to conduct research was obtained from the School of Dentistry, the UWI Campus Ethics Committee and the Public Health Department, EWMSC (Ref: CEC771/11/18) via an Exemption from Review obtained on 14/11/2018. The total number of patients referred from the CDHU for GA/sedation services was noted from the daily emergency clinical log for a 6-year period from 2012 to 2017. The corresponding patient records were then requested, evaluated, and followed up by telephone interviews with parents for those qualifying subjects.

Once informed consent for participation in the study was obtained, a series of open- and close-ended questions were asked. The data collected included age, gender, ethnicity, source and reason for referral, date of referral, time taken to receive treatment or still awaiting the need for treatment, location of treatment, reasons for not receiving treatment, and the need for and type of treatment sought during wait times.

The assessment of the CDHU Emergency clinical log revealed that a total of 420 patients were reportedly referred for GA/sedation between 2012 and 2017. Clinical log records were unavailable for 7 months of the year 2015; thus, this year was omitted from the study. Seventy-five patients’ records could not be retrieved at the time of data collection; hence, these were also excluded. Of the remaining 345 patients referred, 251 (72.8%) patients were reachable, whereas 94 (27.2%) were unreachable via the telephone contacts provided. Therefore, this study included information for a total of 251 subjects.

All data were collected using a data collection form and stored within the premises of the dental school. Data were formatted using Excel to create a database before exporting to Statistical Package for the Social Sciences (SPSS) version 25.0 software (IBM Corporation, Armonk, NY, USA) for statistical (quantitative) data analysis. Both descriptive and inferential statistics methods were used for data analysis. The Chi-squared test was used to assess the association and statistical significance of the referred patients and various treatment outcomes. The level of significance was set at *p* < 0.5.

## 3. Results

Four hundred and twenty (420) (4.8%) of the total number of emergency cases (8790) seen through the CDHU emergency department were referred for treatment under GA/sedation during the aforementioned period (Table 1).

It was noted that there was an average of 28.3% of patients per year, who we were unable to contact via the telephone numbers provided in their patient records (Table 2).

**Figure 1:**
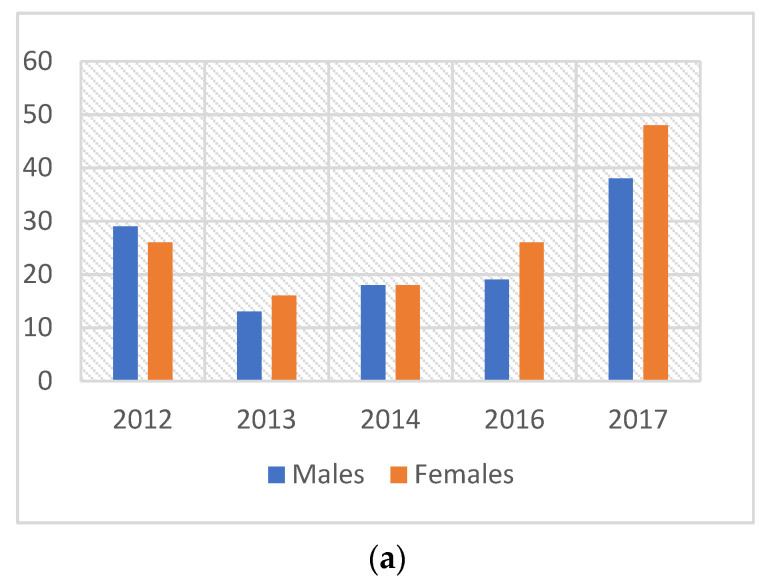

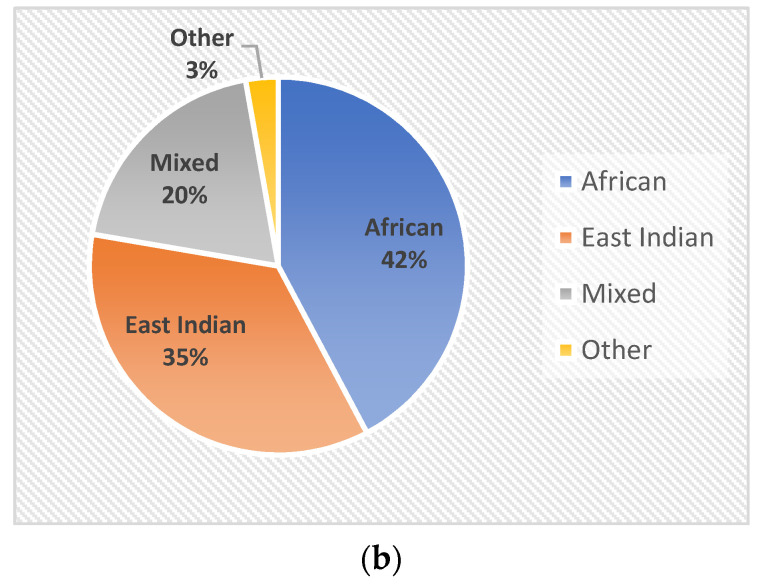
(**a**) Graph showing the average gender distribution of referred patients over the 5-year period. (**b**) Chart representing the overall ethnicity of the referred GA/sedation patient population (%).

Of the 251 children included in this study, 117 (46.6%) were males and 134 (53.4%) were females. The gender distribution per year is illustrated in Figure 1a. The majority of the study population consisted of 107 (42.8%) children of African descent and 89 (35.1%) children of East Indian descent (Figure 1b). One hundred and thirty-four (134) (53.4%) children were less than six years old, 104 (41.4%) were between six and eleven years and 13 (5.2%) were twelve years or older. The ages ranged between 1 and 15 years old, with a mean age of 5.9 years (Table 3).

**Figure 2:**
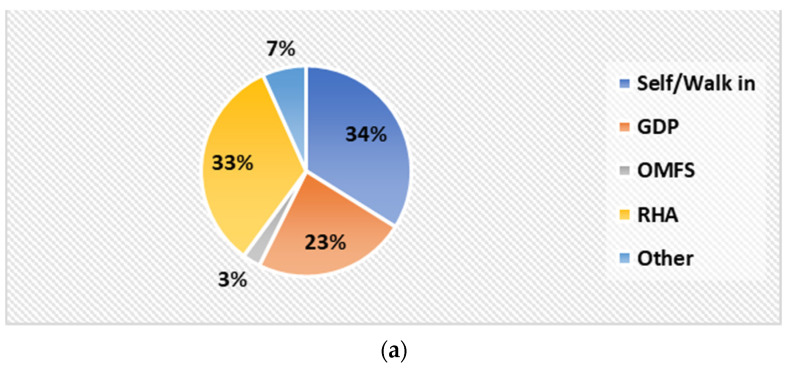

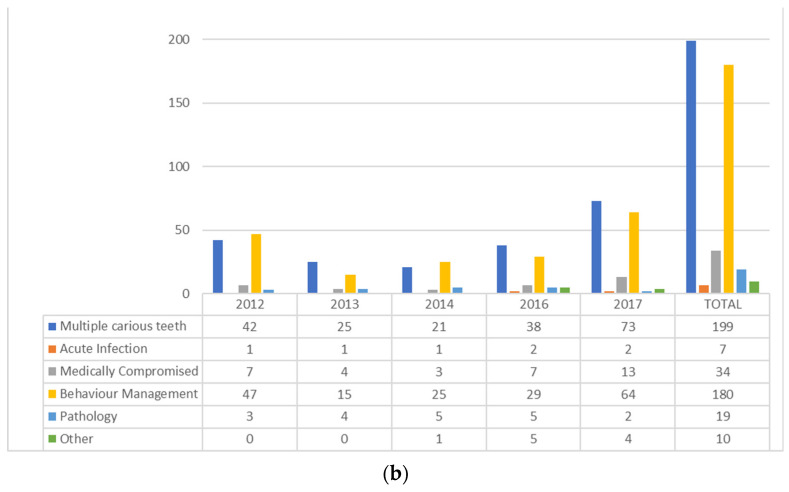
(**a**) Source of referral to CDHU (%); (**b**) chart representing reasons for referral for GA/sedation. GDP—general dental practitioner; OMFS—oral and maxillofacial surgery; RHA—Regional Health Authority.

One hundred and sixty-eight (168) or 66.9% of subjects included were either self-referred (34%) or referred by the government Regional Health Authorities (RHA) community clinics (33%). The most common reasons for referring patients after examination from CDHU emergency clinics for GA/sedation were due to multiple carious teeth (MCT), followed by the need for behaviour management (BM). This consisted of one hundred and ninety nine (199) and one hundred and eighty (180) children, respectively (Figure 2a,b).

### Treatment Outcomes

Details of the treatment outcomes of those referred for GA/sedation are shown in Table 4 and Table 5, respectively. Eighty-five of the 251 (33.9%) children referred were reported to have received treatment; however, 166/251 (66.1%) did not receive treatment.

Of the 85 children who received treatment, 39 (45.9%) sought care privately and 41 (48.2%) were treated at EWMSC. This was not found to be statistically significant. The remaining 5 (5.9%) children reportedly received treatment at either other local or foreign institutions. For those children who were reported to have received care at the EWMSC, the average wait time was found to be 12.9 months (393.5 days). Comparatively, those who received care at private institutions had an average wait time of 4.5 months (136.6 days).

Children who did not receive treatment were reported to have an average wait time of 4.7 years. Furthermore, 111/166 (66.8%) of parents reported cost as the major barrier to receiving care privately. The issue of cost was found to be statistically significant. Moreover, 58/166 (34.9%) parents indicated that their child was no longer in pain; hence, no treatment was eventually sought. Other reasons for not receiving care included never being contacted for either GA consultation/pre-assessment or non-receipt of a confirmed theatre date for treatment at the EWMSC after pre-assessment was undertaken.

The parents of 155/251 (61.7%) children reported that their child needed urgent dental care during waiting times for GA/Sedation, with 46.6% seeking care most often at the EWMSC via CDHU emergency/RHA clinics. The need for treatment during waiting times and seeking this care at the EWMSC during that time were found to be statistically significant. Details of treatment modalities sought during waiting times were shown in Table 5. The treatment modalities sought included temporary restorations with Intermediate Restorative Material (IRM^®^) (38 (15.1%), antibiotics (39 (15.5%)), extractions under LA (45 (17.9%)), analgesics (23(9.2%)), and various preventive regimes. However, the majority of cases (94/251) (37.5%) required re- referrals for GA/sedation. Forty-seven (47) re-referrals occurred within 1 year of the initial referral date and 48 occurred at 1 year or later, with a total number of 157 re-referrals, as some patients had multiple re-referrals.

## 4. Discussion

The results of this study indicate that 4.7% of the patients examined at the CDHU Emergency clinic were referred for GA/sedation for various reasons. The main reasons for referrals were due to multiple carious teeth and the need for behaviour management due to dental anxiety and/or age. This study also indicated that the majority of patients referred for treatment under GA/sedation had a mean age of 5.9 years. These findings were consistent with other reports examining the treatment and outcomes of children receiving GA [3,5,7,12,13,14].

A higher proportion of females (53.4%) in this study, although not statistically significant, were referred for GA/sedation compared to other studies, which reported more males being referred for treatment [15]. However, some studies have reported little to no difference in gender distribution [3,7].

Patients referred from the CDHU for GA/sedation included 34% of self-referrals, 33% who were referred from Regional Health Authorities’ community dental clinics, and 23.5% who were referred by general dental practitioners. Contrasting findings were reported in another study conducted in the UK, where most of their referrals for treatment under GA were initially from general dental practitioners (76.0%), self-referrals (17.1%), and referrals from community clinics (3.7%) [5].

In this setting, this trend can possibly be explained by differences in accessibility to affordable dental care. In the UK, dental treatment of children occurs under primary care dentists or paediatric specialists at community facilities/hospitals when required, at no cost to the parent [16]. Locally, the access to dental treatment by a general dental practitioner or specialist may be financially challenging for some. Individuals may therefore be more inclined to initially seek care at a facility where treatment is provided at little to no cost. These include the CDHU or Regional Health Authority Community dental centres, where access to advanced or specialist dental care is extremely limited.

Compared to studies in other countries, where it was reported that average waiting times for GA/sedation were between 21–137 days [9,15,17,18], in this study the average waiting times for those who received treatment either at the EWMSC regional hospital or private/other institutions were 394 days (12.9 months) and 137 days (4.5 months), respectively. Conversely, for the 66.1% of patients who did not receive treatment under GA/Sedation since their referral date, an average wait time of 4.7 years was observed.

A general delay in receiving dental treatment often results in the development of pain, infection, the disruption of eating and sleeping patterns, and missed school days, which can impact the patients’ and parents’ quality of life [8,17,18,19]. This was reflected by the significant number of referred GA/sedation patients who required emergency care while they awaited treatment and the number of patients who were re-referred for treatment under GA/sedation. The long duration of the waiting time for GA/sedation caused almost one fifth of patients to eventually receive emergency dental extractions under local anaesthesia. This in itself can also exacerbate dental anxiety in the young patient, contrary to the initial recommendation in favour of general anaesthesia.

The use of antibiotics in some children while awaiting treatment is also of concern. The findings in this study appear consistent with the practice of dentists prescribing antibiotics when there is a paediatric dental emergency such as dental abscesses, when cooperation for treatment under local anaesthesia is limited, and when there are anticipated delays in receiving treatment under general anaesthesia [9,20]. This may in turn lead to issues with antibiotic overuse and problems with antimicrobial resistance [21].

The comparative use of oral hygiene, dietary counselling and preventive adjuncts for caries such as fluoride during the wait for general anaesthesia is encouraging, as these non- invasive measures can not only have a positive impact on patient behaviour but can also limit the need for repeat general anaesthesia in these cases [22].

The significant delay in receiving treatment can be due to a variety of patient-related and institution-related factors [14,22,23]. Limited access to affordable GA/sedation services for children was reported to be the main barrier for many parents. The cost of seeking care privately was considered prohibitive by many parents. The access to treatment at the EWMSC regional hospital or other RHAs was either non-existent or limited due to the lack of both human and physical resources. Inability to contact the patient, inadequate staffing to provide advanced dental care, dependency on OMFS to provide GA/sedation services, and the rationing of operating theatre time between OMFS and the various other medical units within the hospital setting were some of the possible reasons for the failure to follow-up on GA/sedation referrals at EWMSC for continued treatment. There may also be a prioritization of patients with pathology such as oral cancer or severe traumatic injuries compared to exodontia of carious teeth unless acute and severe infection developed.

This study had a number of limitations. The study utilized the results of an audit to provide preliminary data and therefore the findings should be generalized with caution in this context. The majority of data for one of the years was not available and thus this year had to be omitted. The socioeconomic status of the parents in this study was not assessed due to a general reluctance of parents to share such information. The type of treatment received under GA/sedation was not investigated, as it was difficult to determine if patients received comprehensive care at private institutions under GA/sedation or if their treatment was limited to addressing emergency concerns, i.e., extractions only.

## 5. Conclusions

A small percentage of patients seen in CDHU were referred for GA/sedation. The majority of cases were females and young children with multiple carious teeth and challenges with behaviour management. Most of the patients referred for GA/sedation did not receive treatment. A lack of appointments at the regional EWMSC hospital and high costs for private care were the main barriers to receiving care. There was a need for dental treatment and the re-referral of more than half of the patients initially referred during their wait times for GA/sedation.

## 6. Practical Applications

Based on the data reviewed in this study, it is the authors’ recommendation that in order to improve the services at the regional hospital and limit the effects of long wait times for treatment under GA/sedation at the institution, the following should be encouraged [18,22,23]:

- A greater focus on the diagnosis and prevention of dental caries at a primary care level to limit the effects of dental caries, especially in younger patients.
- Increased access to GA/sedation services by increasing the number of hospital sites and theatre sessions available for paediatric dental care throughout Trinidad and Tobago.
- Consideration can also be given to implementing a subsidized fee attached to the service provided, perhaps increasing accessibility to many and limiting financial costs to the patients.
- The institution of a recall system to review patients who are referred for GA/sedation to ensure the timely delivery of care.
- The use of non-/minimally invasive treatment modalities can be employed, e.g., oral hygiene and diet counselling, prophylaxis, temporary fillings, and the use of preventive agents such as silver diamine fluoride/Halls crowns to limit the progression of initial/small carious lesions during the wait period for GA/sedation.

## Figures and Tables

**Table 1:** Number of patients seen at the CDHU emergency clinic and the percentage referred for GA/sedation services by year.

	Year				
	2012	2013	2014	2016	2017
Total No. of patients seen	2062	1352	1600	1904	1872
No. referred for GA/sedation Services	107	53	64	74	122
% referred for GA/sedation Services	5.2	3.9	4	3.9	6.5

**Table 2:** Percentage (%) of reachable vs. unreachable patients via telephone contacts provided in patient records by year.

			Year		
*n* = 345	2012	2013	2014	2016	2017
			*n* (%)		
Reachable Patients (251)	55 (66.3%)	29 (67.4%)	36 (70.6%)	45(73.8%)	86 (80.4%)
Unreachable Patients (94)	28 (33.7%)	14 (32.6%)	15 (29.4%)	16 (26.2%)	21(19.6%)

**Table 3:** Age of referred patients by year.

	Year					
Patient Age	2012	2013	2014	2016	2017	Total (%)
<6 years	39	15	20	20	45	134 (53.4%)
6–11 years	14	13	19	18	40	104 (41.4%)
≥12 years	2	1	2	7	1	13 (5.2%)

**Table 4:** Treatment outcomes for patients referred for GA/sedation by year.

	Year						
	2012	2013	2014	2016	2017	Total	*p* Value
	*n* (%)						
**Total No. of Patients referred**	55	29	36	45	86	251	
**Received Treatment**							
YES	24 (43.6%)	9(34.5%)	13 (36.1%)	15 (33.3%)	24 (27.9%)	85 (33.9%)	
NO	31 (56.4%)	20(65.5%)	23 (63.9%)	30 (66.6%)	62 (72.1%)	166 (66.1%)	0.42
**Location treatment was received**							
EWMSC	19 (79.2%)	3 (33.3%)	9 (69%)	3 (20%)	7 (29.2%)	41 (48.2%)	0.002 *
Private	5 (20.8%)	5 (55.6%)	4 (31%)	9 (60%)	16 (66.7%)	39 (45.9%)	
Other	0	1 (11.1%)	0	3 (20%)	1 (4.2%)	5 (5.9%)	
**Reasons for not receiving treatment**							
Cost	0	14 (70%)	19 (82.6%)	26 (86.7%)	37 (59.7%)	96 (57.8%)	0.016 *
No longer experiencing pain	11 (35%)	6 (30%)	10 (43.5%)	10 (33.3%)	21 (33.9%)	58 (34.9%)	
Treatment cancelled/postponed	0	1 (5%)	2 (8.7%)	1 (3.3%)	4 (6.5%)	8 (4.8%)	
Other	24 (77.4%)	5 (25%)	10 (43.5%)	15 (50%)	24 (38.7%)	78 (50%)	
**Need for treatment during waiting times**							
YES	31(56.4%)	21 (72.4%)	22 (28%)	25 (55.6%)	55 (64%)	154 (61.4%)	0.005 *
NO	24(43.6%)	8 (27.6%)	14 (38.9%)	20 (44.4%)	31 (36%)	9 (38.6%)	
**Location of treatment sought during waiting times**							
EWMSC	31(56.4%)	19 (65.5%)	14(38.9%)	19(42.2%)	33 (38.4%)	116 (46.2%)	0.001 *
Private	0	3 (10.3%)	5 (13.9%)	4 (8.9%)	14 (16.3%)	26 (10.4%)	
Other	0	0	6 (16.7%)	7(15.6%)	15 (33.3%)	28 (11.2%)	

* Values were statistically significant (*p* < 0.05).

**Table 5:** Referral and type of treatment received while awaiting GA/sedation services.

Referral Outcome	2012	2013	2014	2016	2017	Total
No. of patients re-referred	17	14	14	16	33	94
Total No. of re-referrals	25	23	33	27	49	157
Re-Referrals needed after 12 months	5	8	10	9	16	48
Re-Referrals needed before 12 months	15	4	4	7	17	47
**Treatment while waiting**						
Oral Hygiene Instruction	9	8	4	6	17	44
Dietary Counselling	8	7	5	4	12	36
Fluoride	6	4	6	6	8	30
Fissure Sealants	1	0	1	4	2	8
Cleanings	2	4	3	5	5	27
Temporary Restorations (IRM^®^)	10	7	5	5	11	38
Antibiotics	9	8	5	8	9	39
Extraction under LA	13	9	4	3	17	46
Analgesics	1	1	4	4	12	22

## Data Availability

The data presented in this study are available on request from the corresponding author.
